# Influence of Resistance Training Variables to Improve Muscle Mass Outcomes in Sarcopenia: A Systematic Review With Meta‐Regressions

**DOI:** 10.1002/jcsm.70162

**Published:** 2025-12-09

**Authors:** Leo Delaire, Aymeric Courtay‐Breuil, Joannès Humblot, Hubert Vidal, Marc Bonnefoy, Emmanuelle Meugnier

**Affiliations:** ^1^ Aging Medicine Department Hôpital Lyon Sud, Hospices Civils de Lyon Oullins‐Pierre Bénite France; ^2^ CarMeN Laboratory, Inserm U1060, Inrae 1397, Université Claude Bernard Lyon 1 Oullins‐Pierre Bénite France; ^3^ RESHAPE Research on Healthcare Professionals and Performance, Inserm U1290, Université Claude Bernard Lyon 1 Lyon France; ^4^ Département des Sciences de l'Activité Physique, Faculté des Sciences Université du Québec à Montréal Montréal Quebec Canada; ^5^ Centre de Recherche de l’Institut Universitaire de Gériatrie de Montréal (CRIUGM) Montréal Québec Canada

**Keywords:** exercise, hypertrophy, muscle, sarcopenia, training

## Abstract

**Background:**

Resistance training (RT) is the first‐line treatment to improve sarcopenia features. However, increasing muscle mass with RT remains challenging and displays inconsistent results. Manipulating training variables may present a novel approach to improve muscle mass gain in sarcopenic individuals. The present study aimed to measure the effectiveness of RT alone on muscle mass outcomes in older adults with sarcopenia and determine the influence of RT variables on muscle mass improvement.

**Method:**

We conducted a systematic review according to PRISMA standards to gather studies that conducted a supervised RT without nutritional intervention in sarcopenia‐diagnosed older adults with a muscle mass outcome versus a control group. A search strategy was performed on PubMed, Medline, Cochrane and Google Scholar in the last 14 years, from the publication of the first agreement on the diagnosis (EWGSOP, 2010). Along with sample characteristics, we extracted and analysed the following training variables: frequency (number of sessions per week), intensity (in rating perceived effort and in % of the one repetition maximal load), duration (weeks), volume (number of sets per week), periodization (yes/no) and muscle failure (yes/no). First, we standardized the outcome with Hedge's *g* and pooled the effect size (ES) of each study in a univariate meta‐analysis adjusted for risk of bias. Then, we performed training composition comparisons between ‘effective interventions’ and ‘ineffective interventions’, which were previously classified based on the 95% confidence interval (CI) effect size. Finally, relevant variables were regressed as moderators of the weighted ES in a mixed‐effects model.

**Results:**

A total of 14 studies representing 528 individuals (73.1 ± 6.6 years, 385 women [73%] and 143 men [27%]) were included for analysis. A significant effect of RT to improve muscle mass was found with a small weighted ES estimate (*g* = 0.38 [0.18; 0.58] 95% CI, *p* ≤ 0.001). There was no publication bias across studies (*p* = 0.7). ‘Ineffective interventions’ included significantly older individuals (*p* ≤ 0.01). Training composition was homogenous between the groups. The final model showed that age was the only significant moderator of the ES (estimate = −0.06 [−0.08; −0.03] 95% CI, *p* ≤ 0.001).

**Conclusion:**

In sarcopenic older adults, designing an evidence‐based RT induces significant gains in muscle mass, but training variables manipulation does not yield greater outcomes. This study also unveils that ageing with sarcopenia negatively affects the significant improvement of muscle mass induced by RT.

## 1 Introduction

Maintaining skeletal muscle mass during the ageing process is critical as muscle tissue not only contributes to force production (with larger fibre diameters inducing stronger muscles) but also plays multiple roles in the organism notably in the hormonal endocrine response (i.e., through myokines secretion), energy production, muscle homeostasis and increases resilience to undesirable events (such as hospitalizations) [1, 2, 3, 4, 5, 6]. Resistance training (RT) is a well‐known key strategy to mitigate the gradual loss of muscle mass and function through ageing [7]. In older adults, it is recognized to be a potent and superior method to increase muscle strength and induce hypertrophy [8]. The main physiological cause underlying muscle myofibrillar hypertrophy with RT relies on the ability of muscles to convert mechanical stimuli into intracellular signalling responses, subsequently increasing net muscle protein synthesis (MPS) accretion by triggering the Mammalian Target of Rapamycin Complexes (i.e., in particular mTORC‐1) [9]. In the context of ageing, RT specifically increases type II muscle fibres cross‐sectional area (CSA) and satellite cell content, thus limiting the age‐related decline in muscle mass [10, 11]. In addition, progressive RT acts as a strong evidence‐based countermeasure of sarcopenia [12], an age‐related syndrome characterized by low muscle strength and mass, leading to several negative consequences such as depression, poor quality of life, falls, mobility disability and mortality [13, 14, 15, 16, 17]. In sarcopenic individuals, substantial improvements in muscle function are obtained in most cases following exercise interventions alone [18]. Yet, gains in muscle mass are less marked and show much more heterogeneous results compared to benefits in muscle function underpinned by strong evidence [19, 20]. Inconsistent data are reported between systematic reviews and meta‐analyses as some studies reported minor and slight significant changes in outcomes of muscle mass (e.g., +0.3 kg in appendicular skeletal muscle mass [21]), whereas many others were inconclusive [20, 22, 23]. However, it should be outlined that these meta‐analyses were not systematically conducted on RT intervention effects but on exercise programmes in the broad sense.

Multiple nonmodifiable factors (e.g., age‐related impaired acute anabolic signalling, poor satellite cell content and micro‐RNA muscle expression), modifiable factors (e.g., deficit in caloric and protein intake, adverse behaviours such as alcohol consumption or a sedentary lifestyle and medication), and differences in assessment techniques are likely responsible for the heterogeneity of responses in muscle mass outcome [24, 25, 26, 27, 28, 29, 30, 31]. RT variables (frequency, intensity, volume, duration and progressiveness) tailored to individual characteristics at baseline are also strong determinants of muscle hypertrophy and insufficient or unsuitable training design will likely result in fewer outcomes, especially in the case of sarcopenia management [32]. Nevertheless, it remains unclear whether RT variables manipulation may contribute to offset the individual variation in hypertrophy and thus could yield larger gains in muscle mass. In healthy older women, an interesting study showed that nonresponsive individuals to muscle hypertrophy following a RT intervention did not gain muscle mass with an additional increase in training volume [33]. On the other hand, a progressive increase in RT volume has been shown to potentiate muscle hypertrophy [34, 35, 36, 37]. In addition, evidence suggests that manifest increases in muscle mass induced by structural muscle adaptations may be affected by training duration (from 12 to 53 weeks) and high volume [19, 38, 39]. As a dose–response relationship of 10 weekly sets per muscle group has been shown to achieve higher gains in muscle mass, RT volume may be an interesting stressor to induce muscle hypertrophy [40].

However, it remains unknown whether RT volume or other training variables influence muscle mass gains in older adults with sarcopenia. Molecular alterations underlying sarcopenia‐related atrophy may lie in dysregulation in anabolic signalling pathways (i.e., the IGF‐1/PI3K/Akt/mTOR pathway) and abnormal autophagy, satellite cell dysfunction, proteasome perturbations and an imbalance between muscle protein synthesis and breakdown [25, 41, 42, 43, 44]. Emerging evidence suggests a wide spectrum of epigenetic alterations controlling these impairments by changing gene expression and myonuclear transcription [45]. Consequently, it is speculated that sarcopenic individuals might experience increased anabolic resistance to exercise, impeding muscle hypertrophy [42]. In order to enhance the anabolic response in sarcopenic individuals, nutritional interventions (i.e., protein supplementation) combined with RT have been investigated multiple times but display limited efficiency and some inconsistent results [46, 47, 48, 49, 50, 51]. Therefore, it appears interesting to explore other approaches for addressing the heterogeneity of anabolic responses in sarcopenia. Examining how RT variables may influence muscle hypertrophy and which would be relevant to manipulate for older adults with sarcopenia embodies a novel approach with clinical implications in order to design a proper RT programme. This approach is of significance as the configuration of training variables is essential to induce muscular adaptations while ensuring an appropriate and acceptable training overload for older adults with sarcopenia [52, 53, 54]. Moreover, it appears of interest to emphasize the importance of RT alone as the key anabolic stimulus for sarcopenia treatment, whereas nutritional interventions should only be considered concomitantly with RT but not as a sole intervention as it is generally considered ineffective alone [51, 55, 56].

To this end, the present systematic review with meta‐regressions aimed at (1) measuring the effectiveness of RT intervention alone (i.e., without nutritional interventions) on muscle mass in older adults with sarcopenia; (2) comparing RT composition between interventions considered to be ‘effective’ versus ‘ineffective’; and (3) determining the influence of RT variables on muscle mass improvement. Based on the evidence available in the literature, we hypothesized that RT volume influences muscle mass gain in this population. Simultaneously, we discussed training transposition and implications required for tailoring RT in an acceptable way for older adults living with sarcopenia.

## 2 Materials and Methods

### 2.1 Search Strategy

The present systematic review was performed according to the Preferred Reporting Items for Systematic Reviews and Meta‐Analyses (PRISMA) to ensure the quality of the study and transparency of results. The search strategy was conducted in accordance with Population, Intervention, Comparison, Outcome and Study design (PICOS) descriptors. The population studied included apparently healthy older adults (aged ≥ 65 years) with a confirmed sarcopenia (or sarcopenic obesity or osteosarcopenia) diagnosed in accordance with either the European Working Group of Sarcopenia in Older People (EWGSOP 1 or 2) or the Asian Working Group of Sarcopenia (AWGS 1 or 2) [57, 58, 59, 60]. According to EWGSOP1 (2010) and AWGS1 (2014), sarcopenia is confirmed when three criteria are met which are low gait speed (< 0.8 m/s), low grip strength (< 20 and < 30 kg in women and men for EWGSOP1; < 18 and < 27 kg for AWGS1) and low skeletal muscle mass index (SMI) (< 6.42 kg/m^2^ and < 8.87 kg/m^2^ in women and men using Bio‐Impedance Analysis (BIA) and < 5.5 kg/m^2^ and < 7.26 kg/m^2^ using Dual X‐Ray Absorptiometry (DXA) for EWGSOP1; < 5.7 kg/m^2^, 7.2 m^2^ and < 7.0 kg/m^2^ using BIA and < 5.4 kg/m^2^ and < 7.0 kg/m^2^ using DXA for AWGS1). The revised EWGSOP2 (2019) retains low muscle strength (either assessed by grip strength or the five‐chair stand test with cutoff values < 16 kg in women and < 27 kg in men or < 15 s) and low SMI (<5.5 kg/m^2^ and < 7.0 kg/m^2^ in women and men using either BIA or DXA) to confirm sarcopenia. Sarcopenic obesity was defined as the association of both low SMI (using skeletal muscle mass divided by body weight * 100 with specific cutoffs) and excessive body fat mass (> 30% using either BIA or DXA). Osteosarcopenia was defined as the association of a confirmed sarcopenia and osteopenia (T‐score <−1.0). We focused on studies that were conducted in community‐dwelling and extended the search with studies conducted in nursing homes to increase study eligibility. The intervention included structured, group‐based RT programmes using either machines, free weights or elastic bands. Muscle mass assessment techniques included BIA, DXA, Computed Tomography scanner (CT‐scan) or Magnetic Resonance Imaging (MRI). As the reference variable for low muscle mass in sarcopenia diagnosis, the outcome comparator included mean value (± Standard Deviation or SD) of the SMI (in kg/m^2^ or in % in the presence of sarcopenic obesity). In cases where SMI was not reported, a raw measure of either appendicular muscle or lean mass was selected to measure the outcome. We focused on randomized controlled trials with at least one arm of RT intervention (or RT predominant intervention). The arm comparator was a control group that did not undergo any other exercise intervention and maintained their usual physical activities. We selected only supervised RT interventions to ensure that training variables were manipulated and controlled by specialists. Nutritional interventions combined with RT were excluded in order to rule out any possible interference on muscle mass outcomes. The search strategy process was performed in several electronic databases including PubMed, Cochrane Library, Medline and Google Scholar. Grey literature (e.g., abstracts, conference papers and theses) and publications in languages other than English were not considered for study identification. Backward citation tracking was performed to increase studies' eligibility. Efforts were made to contact authors (by e‐mail or social networks) in an attempt to obtain complementary information regarding their RT protocol. Two authors (L.D. and M.B.) independently selected studies to be included. The following query according to inclusion and exclusion criteria was used to retrieve studies sought for with each term searched in the [Title/Abstract] section:

(((‘sarcopenia’ OR ‘sarcopenic’) AND (‘resistance training’ OR ‘strength training’ OR ‘exercise’ OR ‘multicomponent’ OR ‘training’) AND (‘muscle mass’ OR ‘lean mass’ OR ‘muscle volume’ OR ‘muscle thickness’ OR ‘cross‐sectional area’)) NOT (‘supplementation’ OR ‘diet’ OR ‘supplement’)) AND (‘clinical trial’ OR ‘randomized controlled trial’) AND ‘humans’ AND (‘aged’ OR ‘aged, 80 and over’) AND 2010/01/01:2024/12/31 [Date–Publication]).

### 2.2 Study Selection Criteria

After the search strategy, the following criteria to select a study were retained: (1) Intervention is a supervised group‐based RT with at least volume and intensity described; (2) diagnosis standards used to confirm the presence of sarcopenia are clearly exposed; (3) at least one muscle mass outcome is reported and has been assessed by the aforementioned techniques; (4) peer‐reviewed and published in English in the last 14 years, as the first sarcopenia diagnosis agreement was published in 2010 [57]. Exclusion criteria were (1) RT combined with any nutritional intervention (supplementation or counselling); (1) multicomponent exercise programme with minor RT exercises; (3) blood‐flow restricted exercise programmes; (4) whole‐body vibration exercise programmes; (5) intradialytic resistance exercise programmes.

### 2.3 Data Extraction

From the selected studies, we extracted the following general data: first author name and year of publication, country and setting (community/institution), total sample with number of men and women, number of participants in each arm, mean age, mean baseline body mass index (BMI in kg/m^2^), population with sarcopenic obesity or osteosarcopenia (yes/no) and muscle mass assessment technique. We extracted the following RT primary variables: frequency (sessions per week), relative intensity in rating perceived effort (RPE) or in percentage of the 1‐repetition maximal load (% of 1RM), duration (weeks), number of exercises per session and rests between sets or exercise (seconds). We estimated the equivalent relative intensity in % of 1RM or RPE based on the degree of effort corresponding to the intensity set in the study (e.g., an intensity load of 60%–65% is considered to be moderate intensity and corresponds to a 11–12 RPE on the Borg's scale 6–20) [61, 62]. Volume training was calculated as weekly sets (number of sets per session × frequency) in order to equate this variable between groups and draw proper causal inferences [63]. In addition, we reported whether participants were asked to perform repetitions to muscle failure (yes/no). Muscle failure was defined when an individual cannot complete the concentric portion of a given repetition with a full range of motion without deviation from the prescribed form of the exercise, namely the momentary muscle failure [64]. Muscle failure was therefore noted when terms such as ‘momentary muscle failure’, ‘failure’, ‘concentric failure’ or ‘repetition maximal’ were found within the selected studies. Finally, we reported whether training was periodized or not (yes/no). Periodization was defined as an organized training towards different phases with intensity and volume variations [65]. Terms such as ‘periodized’, ‘phases’, ‘scheduled’ or ‘progressive intensity and volume’ were retained to confirm if training was periodized or not. Quantitative data extracted were expressed by mean ± SD or median (interquartile range) based on normality distribution or by number and proportion if qualitative. Results are reported in Table 1.

**TABLE 1:** Comparisons of general characteristics, quantitative and qualitative RT variables between ‘effective interventions’ and ‘ineffective interventions’.

	Effective interventions	Ineffective interventions	Overall	Test statistic
Effect size (*g*)				
Mean (SD)	0.69 ± 0.25	0.07 ± 0.23	0.38 ± 0.40	< 0.001
Q1, Q3	0.62, 0.77	0.00, 0.24	0.07, 0.63	
Min, max	0.30, 1.14	−0.35, 0.31	−0.35, 1.14	
Age (years)				
Mean (SD)	68.7 ± 3.5	77.5 ± 6.0	73.1 ± 6.6	< 0.01
Q1, Q3	66.7, 71.0	72.9, 80.9	67.8, 78.6	
Min, max	64.3, 73.7	69.2, 85.9	64.3, 85.9	
BMI (kg/m^2^)				
Mean (SD)	25.7 ± 3.0	27.2 ± 2.5	26.5 ± 2.8	0.3
Q1, Q3	22.6, 28.0	25.9, 28.1	23.7, 28.1	
Min, max	22.4, 28.6	23.3, 31.3	22.4, 31.3	
Females proportion				
Median (IQR)	88.9 (32.5)	75.0 (38.1)	86.1 (41.7)	0.9
Q1, Q3	67.5, 100.0	61.9, 100.0	58.3, 100.0	
Min, max	0.0, 100.0	0.0, 100.0	0.0, 100.0	
Males proportion				
Median (IQR)	11.1 (32.5)	25.0 (42.8)	13.9 (44.0)	0.8
Q1, Q3	0.0, 32.5	0.0, 42.8	0.0, 44.0	
Min, max	0.0, 100.0	0.0, 100.00	0.0, 100.0	
Frequency (sessions/week)				
Median (IQR)	3.0 (1.0)	3.0 (1.0)	3.0 (1.0)	> 0.9
Q1, Q3	2.0, 3.0	2.0, 3.0	2.0, 3.0	
Min, max	2.0, 3.0	2.0, 3.0	2.0, 3.0	
Intensity (RPE)				
Median (IQR)	11.5 (0.2)	11.5 (1.0)	11.5 (0.0)	0.2
Q1, Q3	11.2, 11.5	11.5, 12.5	11.5, 11.5	
Min, max	10.5, 11.5	9.5, 13.0	9.5, 13.0	
Intensity (%1RM)				
Median (IQR)	62.5 (1.5)	62.5 (7.5)	62.5 (2.5)	0.4
Q1, Q3	62.2, 63.7	62.5, 70.0	62.5, 65.0	
Min, max	57.5, 65.0	50.0, 70.5	50.0, 70.5	
Time (weeks)				
Median (IQR)	12.0 (4.0)	12.0 (6.0)	12.0 (3.0)	0.3
Q1, Q3	8.0, 12.0	12.0, 18.0	9.0, 12.0	
Min, max	8.0, 24.0	8.0, 24.0	8.0, 24.0	
Weekly sets volume				
Mean (SD)	50.6 ± 15.0	54.4 ± 20.8	52.5 ± 17.6	0.7
Q1, Q3	45.0, 60.0	40.5, 63.0	38.2, 60.0	
Min, max	24.0, 66.0	30.0, 90.0	24.0, 90.0	
Sarcopenic obesity				
(Row %) (Col %)				> 0.9
No	4 (50.0%) (57.1%)	4 (50.0%) (57.1%)	8 (100.0%) (57.1%)	
Yes	3 (50.0%) (42.9%)	3 (50.0%) (42.9%)	6 (100.0%) (42.9%)	
Muscle failure				
(Row %) (Col %)				> 0.9
No	7 (53.8%) (100.0%)	6 (46.1%) (85.7%)	13 (100.0%) (92.9%)	
Yes	0 (0.0%) (0.0%)	1 (100.0%) (14.3%)	1 (100.0%) (7.1%)	
Periodization				
(Row %) (Col %)				0.4
No	0 (0.0%) (0.0%)	2 (100.0%) (28.5%)	2 (100.0%) (14.3%)	
Yes	7 (58.3%) (100.0%)	5 (41.7%) (71.4%)	12 (100.0%) (85.7%)	

Abbreviations: %1RM, percentage of the 1‐repetition maximum load; BMI, body mass index; *g*, Hedge's *g* effect size; IQR, interquartile range; Q1, Quintile 1; Q3, Quintile 3; RPE, rating perceived effort; SD, standard deviation.

### 2.4 Methodological Quality Assessment and Risk of Bias

**FIGURE 1:**
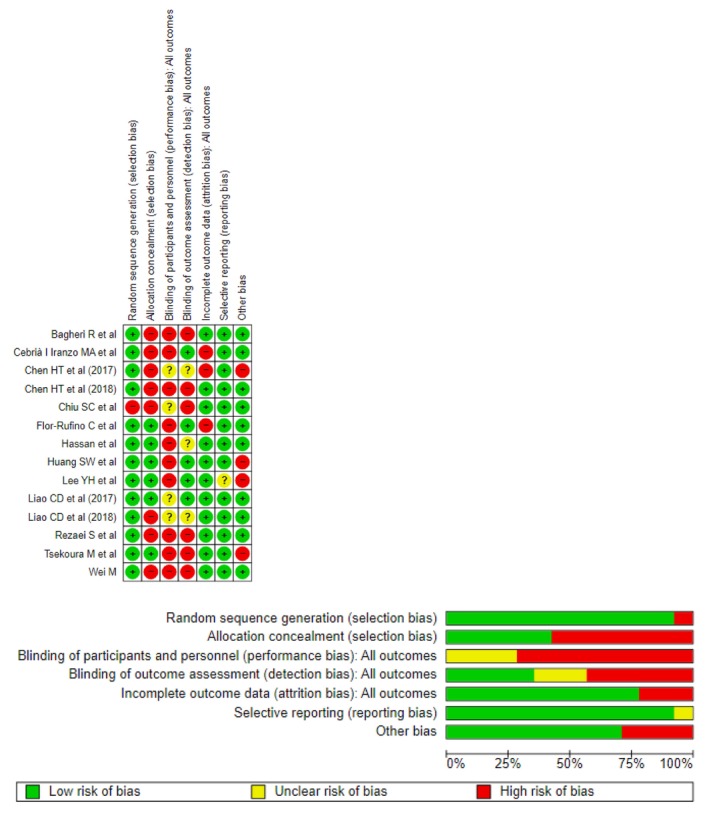
(a, b) The assessment of risk of bias within studies using RevMan Risk of Bias 1. (a) The risk of bias in each study and (b) for each item.

We assessed methodological quality by using the Physiotherapy Evidence Database (PEDro) scale which ranges from 0 to 10 points. The following ranging scores were considered for the quality of the studies selected: 0–3 *poor quality*; 4–5 *fair quality*; 6–8 *good quality*; and 9–10 *excellent quality*. Detail of points' attribution is reported in Table S1. In addition, we measured the risk of bias within studies using the RevMan Cochrane Web tool Risk of Bias 1 (RoB 1). Each bias assessment (i.e., selection, performance, detection, attrition and reporting) was reported in terms of either low, uncertain or high risk of bias. For each study, we also reported the ‘other bias’ regarding the presence of variability measures for the main outcome in muscle mass. Graphical visualization of risk of bias and degree of bias attribution for each item is reported in Figure 1a,b, respectively.

### 2.5 Statistical Analysis

**FIGURE 2:**
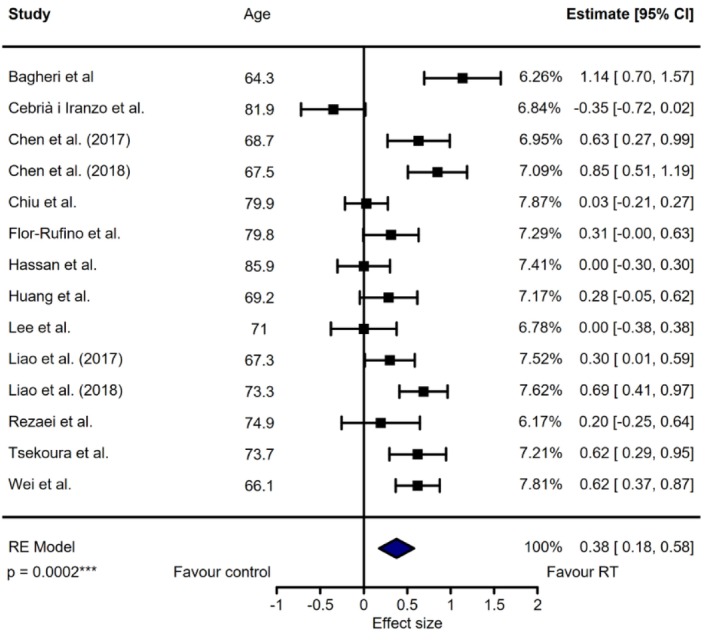
The effect of resistance training interventions on muscle mass outcome resulting from the random‐effects model with a forest plot. Each line represents a study included in the meta‐analysis, and each squared represents their effect size with the 95% confidence interval. The percentage corresponds to each contribution on the weighted effect size.

We measured the effect size between independent samples (i.e., RT intervention vs. control) of each study outcome by Hedge's *g* with a 95% confidence interval (95% CI) due to small samples included. Effect sizes for Hedge's *g* were defined as < 0.2 = trivial, [≥ 0.2; < 0.5] = small; [≥ 0.5; < 0.8] = moderate and ≥ 0.8 = strong. The magnitude of study results was calculated by the 95% CI weighted effect size using a univariate meta‐analysis with a random‐effects model adjusted for potential heterogeneity and publication bias (trim‐and‐fill method) (Figure 2). Heterogeneity measurements were given by tau^2^ (using the restricted maximum likelihood estimator), Cochran's QE test for residual heterogeneity and Higgin's *I* ^2^ tests for total variability. We used a funnel (Figure S1) and a Baujat plot to visualize the residual heterogeneity of the studies included and dispersion around the global effect size. Then, we proceeded to sensitivity analyses by excluding studies that contributed the most to heterogeneity before repeating the meta‐analysis. Assessment of publication bias across studies was measured by Egger's test.

Studies selected were thereafter classified as either ‘effective intervention’ or ‘ineffective intervention’ based on visual inspection of their respective 95% CI effect size on the forest plot. Then, we performed parametric and nonparametric tests for independent samples, as well as Chi‐squared tests for categorical variables, between these two groups. Data analysed were: age, proportion of men and women, BMI, training frequency, training intensity (in RPE and % of 1RM), training duration and weekly sets volume as quantitative variables, and presence of sarcopenic obesity, muscle failure and periodization as qualitative variables. Results are reported in Table 1.

Finally, the aforementioned variables were first regressed using the Least Absolute Shrinkage and Selection Operator (LASSO) method in order to preclude nonrelevant variables (i.e., excluding variables' coefficients equaling zero) and avoid multicollinearity and an overfitted final model. Only the variables kept by the LASSO result were included as potential moderators of the effect size and were thus regressed into the final mixed‐effects model. The omnibus test of moderators was used to measure the significance of moderators in explaining variability between studies and was given by final model results (Table 2). Final model adjustment was checked with residual distribution analyses and adjusted with a robust variance correction meta‐analysis. All statistical analyses and figures were performed under the RStudio environment (Version 4.4.1) for The Comprehensive R Archive Network software (Auckland, New Zealand) using ‘metafor’ (version 4.6) and ‘glmnet’ (Version 4.1.8) libraries. The level of statistical significance was defined as *p* < 0.05 for all test procedures.

**TABLE 2:** Predictors of the effect size.

	Estimate	Standard error	t value	p value	95% CI lower born	95% CI upper born
Intercept	4.24	1.58	2.69	0.03	0.51	7.97
Age	−0.06	0.01	−6.27	<0.001	−0.08	−0.03
BMI	−0.01	0.02	−0.36	0.73	−0.05	0.04
Frequency	−0.15	0.19	−0.77	0.47	−0.61	0.31
Intensity (%1RM)	0.02	0.02	1.22	0.26	−0.02	0.06
Weekly sets volume	−0.01	0.01	−1.53	0.17	−0.02	0.004
Sarcopenic obesity (yes)	−0.07	0.2	−0.43	0.68	−0.47	0.32

Abbreviations: 95% CI, 95% confidence interval; %1RM, percentage of the 1‐repetition maximal load; BMI, body mass index.

## 3 Results

### 3.1 Selected Study Characteristics

**FIGURE 3:**
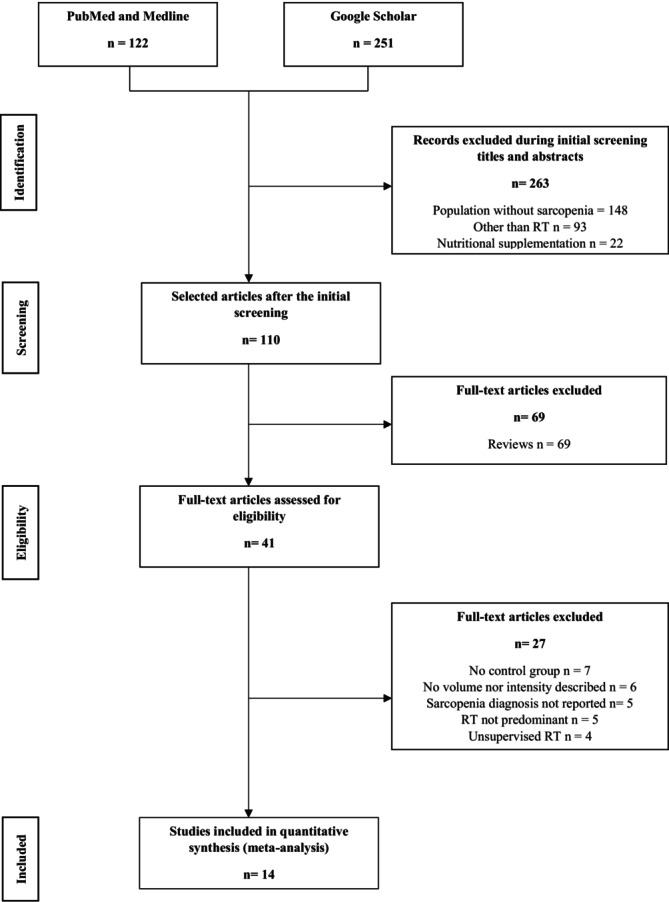
The search strategy and study selection.

The PRISMA diagram flow depicts the search strategy and study selection (Figure 3). A total of fourteen studies were included for analysis which represent a total sample of 528 individuals aged 73.1 ± 6.6 years [66, 67, 68, 69, 70, 71, 72, 73, 74, 75, 76, 77, 78, 79]. Characteristics of the included studies, training composition and results are reported in Table 3. There was a higher proportion of women in the total sample (73%, *p* < 0.0001). Overall, the population included overweight individuals (mean BMI 26.5 ± 2.8); six studies (42.9%) included subjects with sarcopenic obesity among which one included osteosarcopenia and obesity. Most studies were randomized controlled trials (RCT) except for one (i.e., nonrandomized comparative study) that was still included regarding its methodological quality (PEDro score = 6). Eleven studies (78.5%) were designed in community settings, and three (21.4%) were designed in nursing homes. Ten studies (71.4%) were from Asia (mostly Taiwan), three (21.4%) were from Europe and one (7.1%) was from Oceania (Australia). Ten studies (71.4%) applied the EWGSOP diagnosis procedure and three studies (21.4%) applied the AWGS diagnosis procedure. One study applied specific cutoffs to diagnose sarcopenic obesity derived from the Korean National Health Examination Survey cohort [80].

**TABLE 3:** Characteristics and results of the included study.

First author and date of publication	Type of study and time measures	Sample and age	Type of population	Training composition	Assessment techniques of muscle mass and outcomes reported	Results on muscle mass
Bagheri et al. 2020	3 arms Randomized Controlled Trials (RCT) 1 time measure	30 64.3 ± 3.5	Sarcopenic men using EWGSOP with cutoffs for Iranian population Community‐dwelling	F^a^: 3×/weeks I^b^: 40 to 75% of 1RM T^c^: 8 weeks T^d^: machine‐based V^e^: 2× (8–16 reps) for 6 exercises P^f^: ↗ each 2 weeks R^g^: unreported	Bio‐Impedance Analysis (BIA) and blood samples Skeletal Muscle Mass (SMM) Follistatin Myostatin GDF‐11	Both protocols ↑ SMM at 8 weeks and vs. controlled (ET + RT: +0.5 kg; RT + ET: +0.8 kg) as well as ↑ in follistatin, myostatin and GDF‐11
Cebria i Iranzo et al. 2018	3 arms RCT 2 times measures (at 2 and 12 weeks)	37 83.6 ± 6.1	Sarcopenic using EWGSOP with cutoffs for Spanish population Institutionalized	F: 3×/weeks I: 40%–60% 1RM T: 12 weeks T: free‐weights V: 1× 12 reps for 10 exercises P: ↗ each week R: 2′ between sets	BIA Appendicular Skeletal Muscle mass (ASM) ASM/height ASM/weight ASM/BMI	No effect in all measurements
Chen et al. 2017	4 arms blind RCT 2 time measures (8 weeks and 12 weeks)	60 68.8 ± 3.3	Older adults with sarcopenia using NHANES study in Korea and obesity (sarcopenic obesity) Community‐dwelling	F: 2×/weeks I: 60%–70% of 1RM T: 8 weeks T: machine‐based V: 3× (8–12 reps) for 10 exercises P: ↗ each 2 w R: 2 to 3′ between sets	BIA Skeletal Muscle Mass Index (SMI) SMM	RT, AT, and CT had greater SMM & SMI vs. controlled ↗ IGF‐1 in RT and CT only vs. controlled RT not superior than AT to increase muscle mass
Chen et al. 2018	2 arms RCT 2 time measures (8 weeks and after 4 weeks of detraining)	33 67.5 ± 4.1	Sarcopenic women using AWGSOP Community‐dwelling	F: 2×/weeks I: 60%–70% of 1RM T: 8 weeks T: kettlebell‐based V: 3× (8–12 reps) for 11 exercises P: ↗ individualized (when >10 reps were performed) R: 2 to 3′ between sets	BIA SMI SMM ASM	KT ↑ ASM and SMI after 8 weeks (+0.3 kg and +0.1 kg/m^2^) but not SMM KT ↑ SMI vs. controlled*but not SMM and ASM ASM and SMI gains were preserved after detraining
Chiu et al. 2018	2 arms comparative study 1 time measure	64 79.9 ± 7.8	Older adults with sarcopenia using AWGSOP and obesity (sarcopenic obesity) Institutionalized	F: 2×/weeks I: unreported T: 12w T: chair‐based with free‐weight or weight‐bearing V: 3× (4–10 reps) for ~12 exercises P: unreported R: 30 s between sets	BIA SMI ASM Appendicular Skeletal Muscle Index (ASMI)	RT only ↑ SMI at 12 w (+0.8%) but not ASM and ASMI No change of all measurements vs. control
Flor‐Rufino et al. 2023	2 arms blind RCT 1 time measure	38 79.8 ± 7.4	Sarcopenic women using EWGSOP Community‐dwelling	F: 2×/weeks I: 70% of 1RM T: 24w T: machine‐based V: 3× (10–15 reps) until momentary failure for 6 exercises P: unreported R: unreported	BIA and Magnetic Resonance Imaging (MRI) SMI (BIA) Mid‐thigh SMM (MRI) Absolute muscle volume (MRI)	HIRT ↑ SMM and SMI at 6 months and vs. controlled (+1.1 kg; +0.4 kg/m^2^ with moderate effect‐size for both) but not absolute muscle volume HIRT ↑ absolute muscle volume vs. controlled (but was higher at baseline)
Hassan et al. 2016	2 arms RCT	42 85.9 ± 7.5	Institutionalized older adults with sarcopenia using EWGSOP	F: 2×/weeks I: RPE 12–14 (CR6–20) T: 24 weeks T:machine‐based and balance exercises V: 2×–3× (10–15 reps) for 9 exercises P: individually monitored R: unreported	BIA SMI Fat Free Mass (FFM)	No effects in all measurements
Huang et al. 2017	2 arms blind RCT 1 time measure	35 69.2 ± 5.0	Older women with sarcopenia using EWGSOP and obesity (sarcopenic obesity) Community‐dwelling	F: 3×/weeks I: RPE 10–13 (CR6–20) T: 12 weeks T: ERT V: 3× 10 reps for 6 ex P: ↗ when perceived yield strength corresponding to RPE13 was achieved, through full range of motion (ROM) R: unreported	Dual X‐Ray Absorptiometry SMI Total Skeletal Muscle mass (TSM) Muscle mass in arms (left and right), legs (left and right) and trunk	No change in all parameters at 12 weeks and vs. controlled, except for trunk muscle (+0.4 kg)
Lee et al. 2021	2 arms RCT 2 time measures (12 weeks and 6 months after)	27 71.0 ± 4.8	Older women with sarcopenia using EWGSOP, osteopenia and obesity (osteosarcopenic obesity) Community‐dwelling	F: 3×/weeks I: RPE 10–13 (CR6–20) T: 12 weeks T: ERT V: 3× 10 reps for 10 exercises P: ↗ when perceived yield strength corresponding to RPE13 was achieved, through full ROM R:unreported	DXA SMI Appendicular Lean Mass Total Lean Mass Index (LMI)	No change in all parameters at 12 weeks and vs. controlled
Liao et al. 2017	2 arms blind RCT	46 67.3 ± 5.2	Sarcopenic or presarcopenic older women using EWGSOP and obesity (sarcopenic obesity) Community‐dwelling	F: 3×/weeks I: RPE 10–13 (CR6–20) T: 12 weeks T: ERT V: 3× 10–20 reps for 6 exercises P: ↗ when perceived yield strength corresponding to RPE13 was achieved, through full ROM R: unreported	DXA Leg Lean Mass (LLM) FFM	ERT ↑ LLM (+0.6 kg) but not FFM at 12 weeks ERT ↑ FFM and LLM vs. controlled
Liao et al. 2018	2 arms blind RCT 2 time measures (12 weeks and 9 months after)	56 67. ± 5.3	Older women with low muscle mass using EWGSOP and obesity (sarcopenic obesity) Community‐dwelling	F: 3×/weeks I: RPE 10–13 (CR6–20) T: 12w T: ERT V: 3× 10–20 reps for 6 exercises P: ↗ when perceived yield strength corresponding to RPE13 was achieved, through full ROM R: unreported	DXA SMI TSM ASM LMI Appendicular Lean Mass Index (ALMI)	ERT ↑ all parameters at 12 w ERT ↑ all parameters vs. controlled at 12 w except SMI ERT ↑ TSM, ALMI and SMI vs. controlled at 9 months
Rezaei et al. 2024	2 arms randomized groups 1 time measure	19 74.9 ± 5	Sarcopenic men using EWGSOP with cutoffs for Turkish population Community‐dwelling	F: 3×/weeks I: 60%–65% of 1RM T: 8 weeks T: TST V: 3× 8–12 RM for 6 exercises P: ↗ 2 weeks R: 1′ between ex	BIA and Calf Circumference (CC) SMI SMM ASM	No effects of TST on SMI, SMM, ASM and CC
Tsekoura et al. 2018	3 arms RCT 2 time measure (12 weeks and 12 weeks after)	54 72.9 ± 7	Sarcopenic older adults using EWGSOP2 (at least probable sarcopenia) Community‐dwelling	F: 2×/weeks I: RPE 10–12 (CR6–20) T: 12 weeks,1 h per session with 15 to 20 min of RT T: RT with free‐weights in a multicomponent exercise programme V: 1× to 2× (8–12 reps) for 8 exercises P: ↗ 4 weeks R:unreported	BIA and CC SMI	↗ in SMI (+0.2) and CC (+0.4) were greater in GB vs. HB and controlled at 12 weeks No change in SMI and CC after RT for HB CC was greater in GB vs. HB at 24 weeks but not SMI
Wei et al. 2022	3 arms RCT 1 time measure	90 66.3 ± 4.0	Sarcopenic using AWGSOP2 Community‐dwelling	F: 3×/weeks I: 40–60 then 60–80 then 70%–85% of 1RM T: 24 weeks T: machine‐based (3 exercises) and ERT (2 exercises) V: 4× (12–20 reps) then 4× (5–12 reps) then 4× (5–8 reps) P: ↗ each 8 weeks R: 2′ to 3′ between sets	CT scans Third lumbar CSA (L3‐CSA) Relative SMI	RT + YJJ and RT ↑ L3‐CSA and SMI SMI at 24 weeks and vs. controlled RT + YJJ yielded greater improvements in L3‐CSA and SMI

^a^ Frequency (sessions per week).

^b^ Intensity (in % of the 1 repetition maximum load or in Rating Perceived Effort scale).

^c^ Time of intervention (in weeks).

^d^ Type of resistance training.

^e^ Volume (sets x number of repetitions per exercise).

^f^ Progressiveness.

^g^ Rest periods (in minutes).

On average, RT intervention was designed around 3.0 (1.0) sessions per week for a total duration of 12.0 (3.0) weeks. Intensity load was prescribed around 62.5 (2.5)% of 1RM, and the RPE was 11.5 (0) on the Borg's scale 6–20. The weekly sets volume calculated was 52.5 ± 17.6 sets per week (Table 1). None of the studies selected reported rest periods between exercises. Mean rest between sets was 150 (30) s, but nine data (64.3%) were missing. Only one study (7.1%) reported that their participants had to achieve repetitions to muscle failure. Periodization processes were noted in eleven studies (78.6%) (Table 1). RT interventions were based on free weights (28.6%, four studies), elastic band (28.6%, four studies) and machines (28.6%, four studies). One study intervention was based on Total Resistance Exercises (TRX) but still fulfilled inclusion criteria and another used a mix of equipment (machine‐based and elastic bands). All interventions were RT‐based except for two. One was a multicomponent exercise intervention (free‐weights RT, balance and flexibility), and another was concurrent training (RT + aerobic training) [66, 78]. Both of these studies were RT dominant and were thus kept in this meta‐analysis. The outcome SMI (or its proxy) was measured primarily by BIA (64.3%, nine studies), followed by DXA (28.6%, four studies) and CT scan (7.1%, one study). Seven studies checked for the presence of adverse effects within their RT protocol, and none reported any other than acute muscle discomfort in the first weeks of training that disappeared after a few days. The outcome in muscle mass SMI was given in twelve studies (85.7%). For two studies, we selected an SMI proxy given by the skeletal muscle mass (SMM, kg) measured by BIA for one and leg lean mass (in kg) measured by DXA for the other.

Included studies displayed an overall good methodological quality (mean PEDro score 6.5 ± 1.2, Table S1). As exercise intervention protocols are generally not designed for allocating participants and personnel blindly, a high risk of bias was found for the performance and allocation concealment bias (~70% and ~60%, respectively) (Figure 1a,b).

### 3.2 Effectiveness of RT Intervention

We found a significant effect of RT intervention compared to control in improving the muscle mass outcome, with a small‐to‐moderate weighted effect size estimate (*g* = 0.38 [0.18, 0.58] 95% CI, *p* ≤ 0.001) (Figure 2). High heterogeneity was found between studies (tau^2^ = 0.12, *I* ^2^ = 81.6%), and Cochran's QE test was significant (*p* ≤ 0.001). Funnel plot visualization showed that six studies explained this heterogeneity and were equally distributed around the weighted effect size (Figure S1). Baujat plot visualization showed that two studies have, at the same time, a strong contribution to the heterogeneity and to the weighted effect size [66, 67]. Bagheri et al. [66] conducted a concurrent training, and Cebrià i Iranzo et al. [67] intervention was conducted in a nursing home including disabled older adults which could explain their respective variability. Additional meta‐analysis performed without these studies did not change the estimated weighted effect size (*g* = 0.38 [0.21; 0.55] 95% CI, *p* ≤ 0.001) but reduced heterogeneity (tau^2^ = 0.06, *I* ^2^ = 70.9%), albeit being still significant (*p* ≤ 0.001). Similarly, the meta‐analysis performed only with studies that have used BIA did not show any change of the estimate (*g* = 0.37 [0.12; 0.62] 95% CI, *p* ≤ 0.001). We found no publication bias regarding Egger's test (*p* = 0.7). Results of the adjusted meta‐analysis for risk of bias estimated that no study was missing on the left side.

### 3.3 RT Composition Comparisons

Following analyses of the 95% CI effect size of each study, seven (50%, mean *g* = 0.69 ± 0.25) RT interventions were considered to be ‘effective’ to improve the outcome, and seven RT interventions (50%, mean *g* = 0.07 ± 0.23) were considered to be ‘ineffective’ (*p* ≤ 0.001). Statistical tests performed reveal that only age was significantly different between these two groups with older individuals in the ‘ineffective’ group (77.5 ± 6.0 years vs. 68.7 ± 3.5 years, *p* ≤ 0.01) (Table 1). Neither quantitative training nor qualitative variables were different between these two groups (Table 1).

### 3.4 Meta‐Regressions

**FIGURE 4:**
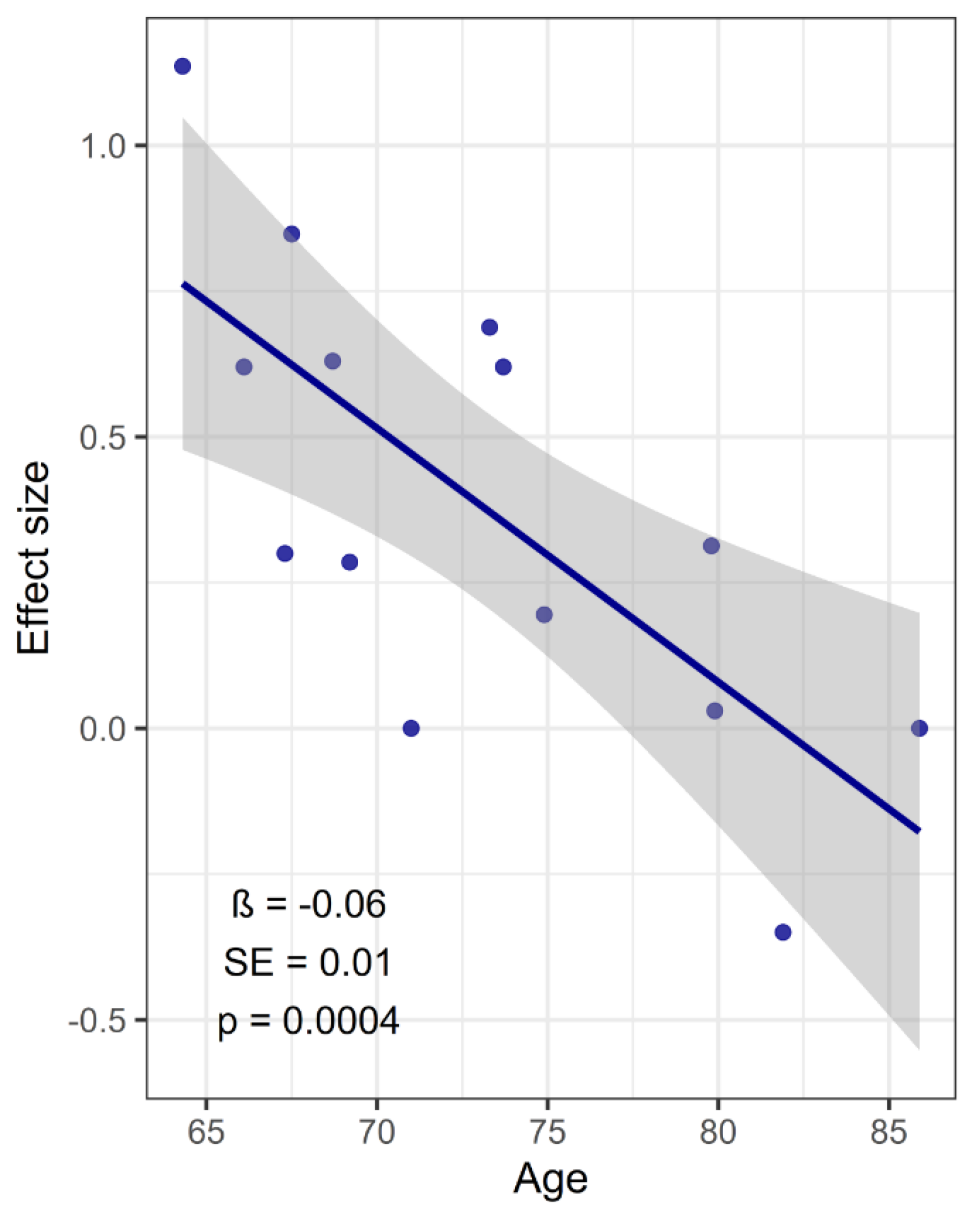
Regression analysis and the effect of age on muscle mass outcome. The continuous blue line represents the regression trend and the dashed line the 95% CI. The text in the lower part mentions the coefficient measure for the variable age its standard error and the *p* value.

The best LASSO regression model with minimum standard error was obtained with six variables (three general and three training‐related) to be included as moderators in the final mixed‐effects model. These variables were age, baseline BMI, presence of sarcopenic obesity, frequency, intensity in % of 1RM and weekly sets volume. Overall, moderators were found to be significant in variability explanation (*p* = 0.005). Results of the final model show five negative and one positive coefficients for moderators (Table 2). Of all the variables regressed, age was the only significant moderator of the effect size (estimate = −0.06 [−0.08; −0.03] 95% CI, *p* ≤ 0.001) (Table 2 and Figure 4). The strongest moderator coefficient was obtained for frequency (estimate = −0.15 [−0.61; 0.31] 95% CI) but was not significant (*p* = 0.5) (Table 2). QE test for residual heterogeneity was still significant (*p* = 0.01) and a high percentage of unaccounted variability was found (*I* ^2^ = 58.7%). Residual distribution from the final model showed nonnormality (Shapiro–Wilk test, *p* ≤ 0.05). Results of the additional meta‐analysis with robust variance correction still showed a significant negative effect of age and identical estimate (−0.06 [−0.08; −0.03] 95% CI, *p* ≤ 0.001) (Table 2).

## 4 Discussion

This study demonstrates that muscle mass improvement following evidence‐based RT protocols in sarcopenic individuals appears to be obtained independently of training variable modifications and was only negatively influenced by age. In addition, this study found that RT led to a significant increase in muscle mass compared to control groups, with a small‐to‐moderate global effect size. This reinforces the importance of progressive RT to struggle against sarcopenia as benefits are not only limited to muscle function improvement, but also to muscle mass [8, 81, 82].

Contrary to the initial assumption, RT volume did not significantly influence the gain in muscle mass in older adults with sarcopenia. As a matter of fact, manipulating RT volume to induce greater outcomes in muscle mass displays discordant results and limited efficacy in healthy older adults. On the one hand, experimental studies have shown that participants exerting at higher volume (three to six sets) had higher gains in muscle or lean mass compared to those exerting at lower volume who exhibited comparable gains (between one and three sets) [34, 36, 83]. This may translate to higher phosphorylation of the downstream regulators of mTORC‐1 (i.e., p70S6 kinase and ribosomal protein S6 complexes) with higher volume as shown in young adults [25, 84, 85]. On the other hand, systematic reviews and meta‐analyses did not conclude any superior effect of high training volume to enhance muscle mass gains [86, 87, 88]. Similarly, Borde et al. [38] found that training volume did not predict the effects of RT on muscle morphology which is consistent with our results. Of note, studies that highlighted greater gains with higher volume were provided with a relatively high number of sets (i.e., three to six sets per exercise). This volume prescription is quite uncommon for older adults with sarcopenia as it is generally more around one to three sets [53]. Exceeding the usual RT volume prescription may lead to higher myofibrillar damage in older adults as revealed by one experimental study conducted in older versus younger women who performed five sets of 11 repetitions on the same muscle group [89]. Moreover, older adults may experience prolonged time of muscle regeneration after an acute metabolic stress such as RT due to a greater amino acid extraction from the splanchnic region, which decreases availability for skeletal muscle [90, 91]. This phenomenon is more specifically expressed in individuals with chronic diseases and the subsequent polypharmacy, both being commonly related to sarcopenia [91, 92, 93]. Therefore, it is argued that prescribing RT volume beyond actual recommendations may exaggerate stress‐induced exercise and may hinder muscle repair capacity, the latter being already impaired with ageing due to satellite cell dysregulation and altered MPS responses [94]. Although being nonsignificant, this may partially explain negative coefficients for training variables weekly sets volume and frequency. In a similar way, increasing training frequency beyond recommendations (> 3 sessions per week) could subsequently result in a higher weekly training volume and may result in less muscle mass gains. This assumption is supported by one study in older adults with sarcopenia who underwent RT combined with aerobic training four times a week and did not observe any improvements in their muscle mass endpoints assessed by MRI [95]. Similarly, comparable gains in SMM were obtained following an RT intervention in older adults with sarcopenia conducted either two or three times a week [96]. As a result, it appears that increasing training frequency or volume beyond recommendations (more than three sets) is not a suitable strategy to yield greater gains in muscle mass in older adults with sarcopenia. Finally, RT intensity did not influence the gain in muscle mass albeit our result suggests a marginal effect of a progressive increase in intensity. This result may be consistent with two meta‐analyses in which authors demonstrated that the best improvement in appendicular SMM with RT in sarcopenic older adults was obtained with a moderate‐to‐vigorous intensity [82, 97].

This study demonstrates that advancing in age negatively affects the outcome of muscle mass after a RT intervention. Compared to young individuals, evidence suggests that the anabolic response to RT is blunted from approximately 60 years and continues to be attenuated with limited adaptive responses at ≥ 75 years [98, 99, 100]. Indeed, a meta‐analysis performed in 452 older adults with a mean age of ~80 years showed no improvement in appendicular muscle mass following RT, which is consistent with this meta‐analysis [101]. In our study, we can notice that all RT interventions conducted in oldest old individuals (i.e., ≥ 80 years) did not induce any improvement in muscle mass [67, 70, 72]. Moreover, these interventions were conducted in nursing homes, a setting where no muscle mass improvement has been shown (with a mean age of 82.6 ± 6.2 years) [102]. At the molecular level, this age‐related anabolic resistance to RT may translate into a further decrease of MPS in response to exercise and amino acid availability, likewise a reduced activation of intracellular anabolic mediators [25, 90, 103]. Interestingly, a comparative study between young (19–30 years) and older adults (76–80 years) demonstrated an upregulation of the protein regulated for development in DNA damage responses 1 (known as REDD‐1) after a RT protocol which blunts m‐TORC‐1 activation [104]. Similarly, Rivas et al. [28] highlighted that one particular micro‐RNA (miR‐19b‐3p) was downregulated in older adults (mean age 77.5 years) who experienced a decrease in leg lean mass following a progressive RT. However, it was demonstrated that following RT, sexagenarians (~64 years) experienced a significant increase in Type II myofibre CSA, albeit with a lower response compared to younger subjects, whereas the expression of myogenic differentiation factors was similar between the groups [105]. In light of the above‐mentioned evidence, age‐related anabolic resistance to RT may progress with ageing. This argument is supported by Slivka et al.'s [106] work, who concluded that, as opposed to septuagenarians, gains in knee maximal isometric strength in octogenarians after RT were primarily neuromuscular‐driven, as the marginal increase in thigh CSA was not associated with an increase in type IIa fibre diameters nor in a shift in myosin heavy chain expression. However, these assumptions are contrary to Ahtiainen et al.'s [107] retrospective study who showed that, in a cohort of 287 subjects, post‐RT gains in muscle size were induced regardless of age subjects ranging from 19 to 78 years who were healthy and nonsarcopenic. Similarly, healthy older adults separated into two groups (65–75 years vs. ≥ 85 years) obtained similar gains in muscle mass following a 12‐week RT [108]. As a result, it appears that ageing with sarcopenia is seemingly deleterious for improving muscle mass. As ageing is associated with increased anabolic resistance, the presence of an associated sarcopenia could be a factor exacerbating this physiological phenomenon particularly in the oldest old [42, 84, 90, 109]. This hypothesis may be supported by Lu et al.'s [110] study who demonstrated that sexagenarians with sarcopenia (mean age 69.9 ± 4.7) were as responsive in the SMI as the nonsarcopenic group following a RT intervention. Moreover, evidence from a cross‐sectional study in 3276 older adults reports a reduced concentration of anabolic hormones Insulin‐like Growth Factor‐1 and Growth Hormone in sarcopenic versus nonsarcopenic individuals [111]. In the same study, the authors also found that participants with sarcopenia were significantly older (77.7 ± 8.4 years). Thus, sarcopenia may impede muscle hypertrophy induced by RT only from a certain advanced age, presumably from 75 to 80 years. The underlying mechanisms occurring progressively may include a reduced amino acid availability and utilization resulting in a blunted MPS in response to exercise despite a paradoxical higher mTORC‐1 activation, impaired anabolic signalling from intracellular and extracellular depleted mediators, and lower myonuclear and satellite cell content [24, 42, 90, 91, 112].

The persistent heterogeneity observed indicates that variability between studies may rely on other factors. Although these studies did not provide any nutritional intervention, it should be considered a prominent factor in the different muscle mass responses. Indeed, a systematic review with meta‐regressions demonstrated that, in adults, a deficit of 500 kcal per day impairs gains in lean mass during RT [26]. Actual international recommendations for older adults engaging in exercise programmes indicate a minimum of 1.2 g·kg/day protein intake to preserve lean mass but higher doses (1.5–1.6 g·kg/day), specific proteins (i.e., in particular rich in leucine and whey), and timing ingestion may be required for older adults with sarcopenia to stimulate MPS to a greater extent [113, 114, 115]. The maintenance of regular physical activities between supervised sessions may also play a role as suggested by one study [116]. In addition, preliminary data suggest that individuals who were previously trained in the past may present a greater muscle plasticity to promote muscle hypertrophy more rapidly [117]. Omics studies identified specific genes upregulated with RT which retained hypomethylation after training cessation suggesting epigenetic memory [118]. However, the exact underlying mechanisms and contributors to muscle memory are still debated [112].

Our study has some limitations. Although the selection procedure was performed independently, no submission on the PROSPERO platform was realized prior to the search strategy. It should also be outlined that ‘effective’ and ‘ineffective’ interventions were relatively homogenous in training variables which may have decreased model sensibility to detect a potential influence. The results were provided with a relatively low number of studies and a persistent residual heterogeneity was present despite the significance of moderators. Finally, it is worth mentioning that the discrepancies across assessment techniques in measurement accuracy, specificity (i.e., lean or muscle mass being measured) and feasibility/accessibility limit results comparability. In this context, the authors have also suggested shifting from the traditional minimal detectable changes to highlight what is clinically meaningful in terms of muscle mass responses [119].

This study presents several strengths. First, meta‐analysis results were not changed despite the variability between studies. Sensitivity analysis performed did not alter the estimate. Similarly, meta‐regression results and the effect of age were not altered by the nonnormality of final model residuals. By excluding nutritional interventions, we provided strong evidence (despite variability between studies) that RT alone induced significant muscle adaptations and improvement in muscle mass, although with an overall small effect size (*g* = 0.38).

## 5 Conclusion

The main finding of this systematic review with meta‐regressions is the negative effect of ageing on the improvement of muscle mass following RT in older adults with sarcopenia. It is hypothesized that sarcopenia may exacerbate the age‐related anabolic resistance possibly due to several molecular alterations that require further investigation. To support this hypothesis, this result should be compared to a similar population without sarcopenia in order to confirm the deleterious aspect of ageing with sarcopenia. To overcome this potential barrier to muscle hypertrophy, strategies focusing concomitantly on diet management and physical activity maintenance could be of interest. Conversely, RT variables did not have a significant influence on the muscle mass outcome. Designing an evidence‐based RT appears to be sufficient to induce muscle hypertrophy in sarcopenic individuals and manipulating training variables does not result in greater outcomes. In oldest old sarcopenic individuals (75–80 years), it still may be interesting to investigate whether RT prescribed with lower sets and a higher number of repetitions performed at lower intensity (using %1RM load) close to failure could be beneficial for muscle hypertrophy, while monitoring progressiveness and tolerance. Training variables variations during RT should be manipulated cautiously in older adults with sarcopenia to find a compromise between adaptations induced by training overload and exercise tolerance. In addition, this study provides strong evidence of the effectiveness of RT alone to improve muscle mass with a small‐to‐moderate effect size. Therefore, RT should be prescribed widely in the context of sarcopenia management. When programming a structured and supervised RT in line with evidence‐based guidelines, healthcare professionals could expect improvements at the same time in strength as well as in muscle mass. Primary prevention strategies including RT programmes should also be implemented at older ages and on a wide scale to the extent that sarcopenia may impede RT‐induced muscle hypertrophy.

## Funding

The authors have nothing to report.

## Ethics Statement

The authors have nothing to report.

## Conflicts of Interest

The authors declare no conflicts of interest.

## Supporting information


**Table S1:** Point's attribution for the calculation of the Physiotherapy Evidence Database scale.

**Figure S1:** depicting the funnel plot of the studies included. The vertical dotted line represents the weighted effect size and each point represents a study distributed based on their risk of bias.
