# Supplementary material for: Influence of Resistance Training Variables to Improve Muscle Mass Outcomes in Sarcopenia: A Systematic Review With Meta‐Regressions
Source: J Cachexia Sarcopenia Muscle. 2025 Dec 9;16(6):e70162. doi: 10.1002/jcsm.70162 (PMC12688407; doi:10.1002/jcsm.70162)
Supplement: Supplementary file 1 — Table S1: Point's attribution for the calculation of the Physiotherapy Evidence Database scale. [file JCSM-16-e70162-s001.docx]

| First author | Inclusion | Randomization | Blind assignation | Comparative groups | Blind subjects | Blind therapists | Blind assessors | 85% of reassessment | Intention-to-treat | Statistical differences | Variability measures | Total score |
| --- | --- | --- | --- | --- | --- | --- | --- | --- | --- | --- | --- | --- |
| Bagheri et al | 1 | 1 | 0 | 1 | 0 | 0 | 0 | 1 | 1 | 1 | 1 | 6 |
| Cebria i Iranzo et al | 1 | 1 | 0 | 1 | 0 | 0 | 1 | 0 | 1 | 1 | 1 | 6 |
| Chen et al (2017) | 1 | 1 | 0 | 1 | 0 | 0 | 1 | 0 | 1 | 1 | 0 | 5 |
| Chen et al (2018) | 1 | 1 | 0 | 1 | 0 | 0 | 0 | 1 | 1 | 1 | 1 | 6 |
| Chiu et al | 1 | 0 | 0 | 1 | 0 | 1 | 0 | 1 | 1 | 1 | 1 | 6 |
| Flor-Rufino et al | 1 | 1 | 1 | 1 | 0 | 0 | 1 | 0 | 1 | 1 | 1 | 7 |
| Hassan et al | 1 | 1 | 1 | 1 | 0 | 0 | 0 | 1 | 1 | 1 | 1 | 7 |
| Huang et al | 1 | 1 | 1 | 1 | 0 | 0 | 1 | 1 | 1 | 1 | 0 | 7 |
| Lee et al | 1 | 1 | 1 | 1 | 0 | 0 | 1 | 1 | 1 | 1 | 0 | 7 |
| Liao et al (2017) | 1 | 1 | 1 | 1 | 1 | 1 | 1 | 1 | 1 | 1 | 1 | 10 |
| Liao et al (2018) | 1 | 1 | 0 | 0 | 0 | 1 | 1 | 1 | 1 | 1 | 1 | 7 |
| Rezaei et al | 1 | 1 | 0 | 0 | 0 | 0 | 0 | 1 | 1 | 1 | 1 | 5 |
| Tsekoura et al | 1 | 1 | 1 | 1 | 0 | 0 | 0 | 1 | 1 | 1 | 0 | 6 |
| Wei et al | 1 | 1 | 0 | 1 | 0 | 0 | 0 | 1 | 1 | 1 | 1 | 6 |

**Table S2. Point’s attribution for the calculation of the Physiotherapy Evidence Database scale.**
