# Supplementary material for: Influence of Resistance Training Variables to Improve Muscle Mass Outcomes in Sarcopenia: A Systematic Review With Meta‐Regressions
Source: J Cachexia Sarcopenia Muscle. 2025 Dec 9;16(6):e70162. doi: 10.1002/jcsm.70162 (PMC12688407; doi:10.1002/jcsm.70162)
Supplement: Supplementary file 2 — Figure S1: depicting the funnel plot of the studies included. The vertical dotted line represents the weighted effect size and each point represents a study distributed based on their risk of bias. [file JCSM-16-e70162-s002.pdf]

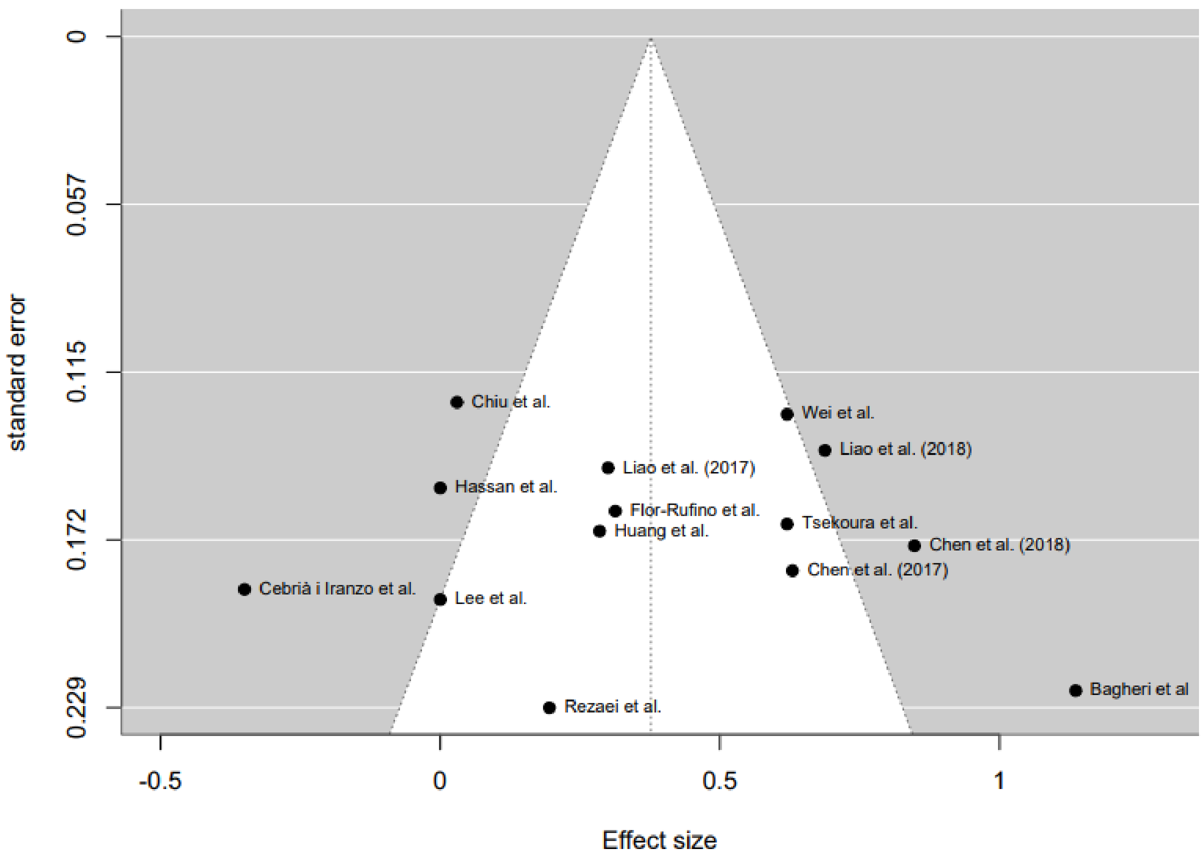

Fig. S1 depicting the funnel plot of the studies included. The vertical dotted line represents the weighted effect size and each point represents a study distributed based on their risk of bias.

Influence of resistance training variables to improve muscle mass outcome in sarcopenia: a systematic review with meta-regressions

Journal of Cachexia, Sarcopenia and Muscle wasting

Leo Delaire[1,2], Aymeric Courtay-Breuil[1,3,4], Joannès Humblot[1], Hubert Vidal[2] , Marc Bonnefoy[1,2] , Emmanuelle Meugnier[2]

[1] Aging Medicine Department, Hôpital Lyon Sud, Hospices Civils de Lyon, Oullins-Pierre Bénite, France.

[2] CarMeN Laboratory, Inserm U1060, Inrae 1397, Université Claude Bernard Lyon 1, Oullins-Pierre Bénite, France.

[3] RESHAPE Research on Healthcare Professionals and Performance, Inserm U1290, Université Claude Bernard Lyon 1, Lyon, France.

[4] Université du Québec à Montréal, Faculty of Science, Department of Physical Activity Sciences, Montréal, Qc, Canada.

Corresponding author: Leo Delaire ; leo.delaire@chu-lyon.fr ; +33 6 13 96 65 59.
